# Cross-cultural survey development: The Colon Cancer Screening Behaviors Survey for South Asian populations

**DOI:** 10.1186/s13104-017-3098-3

**Published:** 2017-12-28

**Authors:** Joanne Crawford, Dorcas Beaton, Farah Ahmad, Arlene S. Bierman

**Affiliations:** 1 Faculty of Applied Health Sciences, Department of Nursing, Brock University, 1812 Sir Isaac Brock Way, St. Catharines, ON L2S 3A1 Canada; 2 Musculoskeletal Health and Outcomes Research, Li Ka Shing Knowledge Institute, St. Michael’s Hospital, Toronto, ON Canada; 3 Measurement Stream, Institute for Work & Health, Toronto, ON Canada; 4 Department of Occupational Science and Occupational Therapy, Rehabilitation Sciences Institute, and the Institute of Health Policy, Management and Evaluation, University of Toronto, Toronto, ON Canada; 5 School of Health Policy and Management, Faculty of Health, York University, 4700 Keele Street, Toronto, ON Canada; 6 Center for Evidence and Practice Improvement (CEPI), Agency for Health Care Research and Quality, Washington DC, USA; 7 University of Toronto, Toronto, ON Canada

**Keywords:** Early detection of cancer, Colorectal cancer screening, Health behaviours, South Asian, Survey, Measurement

## Abstract

**Objective:**

The objective of this work was to develop a survey that considered cultural relevance and diversity of South Asian populations, with the aim of describing or predicting factors that influence colorectal cancer screening intention and adherence. The scientifically rigorous approach for survey development informed the final phase of an exploratory mixed method study. This initial survey was later cross-culturally translated and adapted into the Urdu language, and thereafter, items were cognitively tested for conceptual relevance among South Asian immigrants.

**Results:**

The initial development of the Colon Cancer Screening Behaviours Survey for South Asian populations was completed using a number of steps. Development involved: the identification of key concepts and conceptual model; literature search for candidate measures and critical appraisal; and, expert consultation to select relevant measures. Five published surveys included measures that covered concepts relevant to South Asians and colorectal cancer screening behaviours. However, measures from these surveys missed content that emerged through parallel field work with South Asians, and additions were required along with item modifications. In the final stage, cross-cultural translation and adaptation into Urdu, and cognitive testing were completed. Future research will require an examination of proposed relationships, and psychometric testing of measures in the survey.

**Electronic supplementary material:**

The online version of this article (10.1186/s13104-017-3098-3) contains supplementary material, which is available to authorized users.

## Introduction

Globally, colorectal cancer (CRC) is among the highest in North America and Europe [1]. Early detection of CRC using the fecal occult blood test (FOBT) has been shown to reduce relative risk of mortality by 15% if performed biennially compared to no screening [2]. Population-based CRC screening using the FOBT or fecal immunochemical test has been implemented internationally [3]. Yet, CRC screening uptake is low among South Asians (SAs) settled in western countries [4, 5].

South Asian (SA) populations are growing in the United Kingdom (UK), United States of America (USA), and Canada due to increasing global migration. Prior studies report low CRC screening among SAs. For instance, CRC screening was low among SAs compared to non-Asian UK populations, 32.8% versus 61.3%, respectively [4]. In the USA, SAs were less likely to have obtained CRC screening compared to non-Latino Whites, 42.3% and 57.7%, respectively [5]. CRC risk is low among newcomer SAs; however, with time spent in settlement country, risk approaches similar incidence as native-born populations [6–10]. This increase has been linked to post migration factors such as the adoption of westernized lifestyle behaviors [10, 11].

Survey research is used to examine cancer screening behaviours; yet, less attention has been paid to survey development with inclusion of ethno-cultural relevance. Our team developed a survey to examine prevalence, beliefs and attitudes, facilitators and barriers to CRC screening among SAs in the UK, USA, and Canada. Data was drawn from phase one and phase two of a mixed method study [14, 15], and expert consultation [12].

## Main text

### Colon Cancer Screening Behaviours Survey

The survey was developed as part of an exploratory mixed method study conducted in Canada, and underpinned by critical social theory [13] that included a scoping study, focus group study, and survey development and cognitive pre-test study [12]. The scoping and focus group studies [14, 15] formed the basis of initial work to uncover concepts to examine CRC screening behaviours among SAs. The survey was cross-culturally translated and adapted into Urdu, and cognitively pre-tested (English and Urdu) with SAs in Canada [16]. This paper reports on the systematic and scientifically rigorous steps undertaken prior to reaching the final stage with the aim of encouraging comprehensive approaches in the field.

### South Asian populations

SAs are individuals from India, Pakistan, Bangladesh, Sri Lanka, and the SA *diaspora* (i.e. SAs migrating from countries such as South Africa) [17, 18]. Rapidly growing in the west, SAs represent: the third largest Asian group in the USA [19]; the second largest minority group in the UK [20]; and, the first largest minority group in Canada [21]. Scoping and focus group studies [14, 15] elucidated on socio-cultural context of cancer screening among SAs.

### Screening among South Asians

The scoping study reported on SA beliefs, attitudes, and barriers regarding breast, cervical, and CRC screening in the UK, USA, and Canada [14]. Common barriers included: (a) lack of knowledge [22–25]; (b) language barriers [22, 25]; (c) low literacy [23, 26]; (d) low self-perceived risk [22, 27]; and (e) cost and time [23]. Few studies examined SA beliefs and attitudes related to CRC screening, particularly in Canada [23, 26]. Given the uniqueness of CRC screening (i.e. procedures and gender preferences), a focus group study was required.

To examine CRC screening behaviours, focus groups with 42 SAs originating from India, Pakistan, Bangladesh, Mauritus, Uganda, and Kenya were conducted in Canada [15]. The Behavioural Reasoning Theory [28] incorporates behavioural and social context, and guided the interview protocol, which was later pilot tested with SAs. SA research assistants trained to conduct focus groups recruited participants from community settings in Ontario [15]. Findings revealed factors that influenced CRC screening, such as: benefits of early detection; screening was not believed to be necessary; lack of knowledge, and family physician support and access [15]. These collective findings informed our conceptual model.

### Conceptual model

**Fig. 1:**
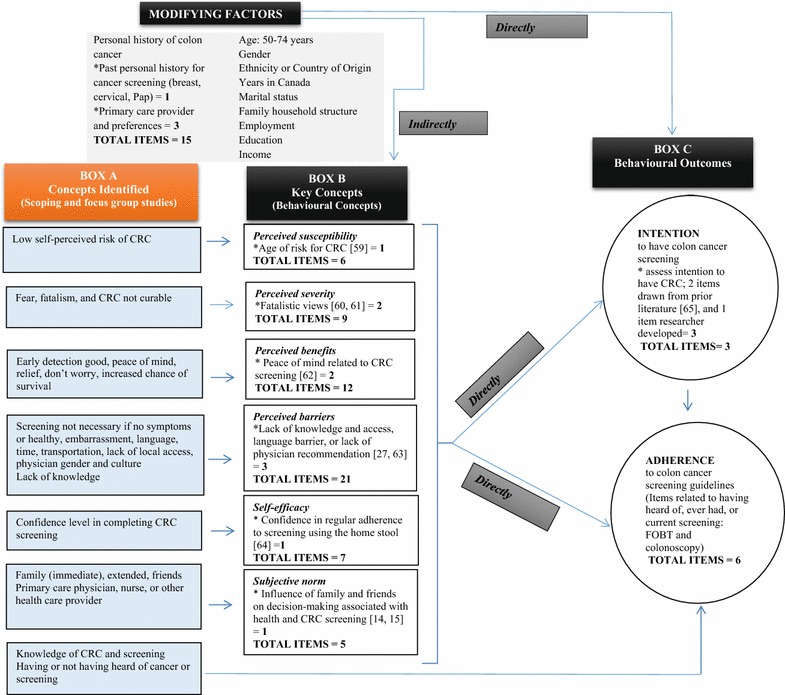
Conceptual model of the Colon Cancer Screening Behaviors Survey. * Denotes an added item to cover missing content identified from the scoping and focus group studies [10, 11]

Key concepts identified from our studies [14, 15] were charted (see Table 1); thereafter, a review of health behaviour theories was conducted to determine conceptual congruence. Behavioural concepts from the Health Belief Model (HBM) [29] and the Theory of Planned Behaviour (TPB) [30] aligned well to our key concepts (see Fig. 1, Box A & B). *Perceived susceptibility* aligned with SAs low perceived risk of cancer [14, 15]. *Perceived severity* reflected SA beliefs that cancer was scary, and had poor outcomes [14, 15]. *Perceived benefits* linked to reduced worry and improved survival with screening [14, 15]. *Perceived barriers* aligned to language and cost barriers [14, 15]. *Perceived self*-*efficacy* related to low confidence with completing the test (i.e. FOBT) [14]. *Subjective norm* reflected the influence of family and physician to have screening [14, 15]. Socio-contextual variables were also considered [31]. The emergent conceptual model is a product of primary research with SAs and existing theoretical literature (see Fig. 1).

**Table 1:** Key concepts identified

Key concepts Scoping study [9]	Key concepts Focus group study [10]	Conceptual definition	Candidate measures*
Low self-perceived risk of CRC Not a risk: Most prevalent in western populations Not necessary—no symptoms	Lack of knowledge of risk Screening not necessary: No symptoms or healthy	*Perceived susceptibility* [HBM] Is concerned with the self-perceived risk of a diagnosis of CRC	Leung et al. [46] Ozsoy et al. [47]
Fear of cancer and diagnosis CRC not curable Consequences of cancer (stigma)	Cancer is scary: Fear associated with test or waiting for results, and diagnosis Consequences of cancer (bring on illness or poor outcomes)	*Perceived severity* [HBM] An individual’s belief of the serious nature of a CRC, its impact (medical, clinical, social, and physical) and evaluation if not treated	Leung et al. [46] Ozsoy et al. [47]
Screening increases chance of survival or cure	Early detection is good Start treatment soon Better survival and cure Screening reduces worry	*Perceived benefits* [HBM] An individual’s belief that a change of behaviour results in a benefit; thus reducing the threat of CRC. This could relate to health and social consequences (i.e. detect polyp early, reduced worry or appease family by doing screening), which can also influence decision-making	Leung et al. [46] Ozsoy et al. [47] Rawl et al. [45]
Lack of knowledge—not heard about cancer, risks or screening, and do not know how to do test Fear, nervous, worry, pain, embarrassment, or unpleasant Language, cost, time, no transportation Lack of physician recommendation	Lack knowledge: Not heard about CRC, risks, or screening Aversion to collecting stool Language of physician	*Perceived barriers* [HBM] Is linked to factors that impede decisions to act by having CRC screening; the pros and cons are weighed	Leung et al. [46] Ozsoy et al. [47] Rawl et al. [45]
Low confidence in completing screening Confidence to do test		*Perceived self*-*efficacy* [HBM] An individual’s confidence in the ability to complete a home stool test or preparation for colonoscopy	Flight et al. [48]
Family as central—provide advice and support Loss of social support Physician recommendation	Family and friends Family physician, nurse practitioner or other HCP Physician recommendation, responsibility, explanation and enforcement	Subjective norm [TPB] The individual’s perception of others expectations of performing CRC screening, and the ability of the individual to comply with others	Flight et al. [48] Ozsoy et al. [47]
Screening for breast, cervical and CRC low among SA immigrants Not heard of or had cancer screening	Not heard of CRC, risks and screening Not had CRC screening (FOBT)	*INTENTION & ADHERENCE* Self-report information that reflects having heard of and/or use of the home stool test and colonoscopy screening. Plans to have CRC screening	Vernon et al. [49]

*Note* Rawl et al. [45] modified *perceived benefits* and *barriers* from Champion’s [50] breast cancer screening measures. Leung et al. [46] and Ozsoy et al. [47] used previously adapted CRC screening measures [32, 43] originally developed as breast cancer screening measures from Champion [51] and Champion and Scott [52]. Flight et al. [48] utilized prior measures drawn from Tiro et al. [53] and Vernon et al. [54] originally based on a number of health behaviour theories including the HBM [29] and TPB [30]. Vernon et al. [49] developed self-report measures for CRC screening awareness and adherence

* papers with measures used emerged from prior studies

The HBM [29] and TPB [30] were used to operationalize conceptual definitions (Table 1). The HBM [29] and the TPB [30] have been incorporated into measures that examined CRC screening among diverse populations [27, 32–34], and a few have combined concepts from both into a single survey [35, 36]. Thus, we were confident in our decision to utilize these behavioural concepts as the best fit to our key concepts.

As depicted in Fig. 1 (Box C), two behavioural outcomes include intention and adherence to CRC screening (Table 1). Intention is a precursor to CRC screening, while adherence is compliance with screening recommendations [37, 38]. According to the HBM [29] and TPB [30], *perceived susceptibility, perceived severity, perceived benefits, perceived barriers, perceived self*-*efficacy,* and *subjective norm* directly influence CRC screening intention and/or adherence [39, 40]. In our conceptual model, key concepts equally influence CRC intention or screening with no direct relationship between concepts specified.

According to the HBM [29], modifying factors indirectly influence behavioural outcomes (see Fig. 1). Screening history and socio-demographics represent socio-cultural context of screening and may directly influence outcomes [14, 15].

### Comprehensive literature search

To identify articles that reported on candidate measures assessing cancer screening, a comprehensive literature search guided by DeVellis framework [41, 42], and librarian recommendation was conducted. Five databases were searched: Ovid Medline [1946 to March week 1 2015], EMBASE [1947 to 2015 March 09], PsychoINFO [1806 to March week 1 2015], CINAHL [1988 to 2015, March 9], and Health and Psychosocial Instruments [1985 to March 2015]. Grey literature search of the UK Bowel Screening Program and Cancer Research UK websites were completed. Reference lists were reviewed.

A combined total of 426 citations were returned. In selecting articles, inclusion and exclusion criteria were applied: (a) availability in English; (b) any cancer screening; (c) examination of beliefs, attitudes, facilitators or barriers using defined measures, and; (d) any population. Duplicates, dissertations, reviews, conference abstracts, and books were excluded. A total of 142 citations remained after applying inclusion and exclusion criteria. Of these, 78 were excluded because they were cross-sectional application studies that used previously developed or adapted measures. The remaining 64 articles reported on newly developed, previously created, and adapted measures; most were initially developed for breast cancer screening and later adapted to assess CRC screening [32, 43–45].

We decided to focus on measures that examined CRC screening because of unique procedures; 24 articles underwent full-text review. A further 19 articles were excluded because measures did not match key concepts or lacked conceptual definitions (Additional file 1: Literature search flow chart).

Five published surveys [45–49] were selected as the best match, and had the most promise because they were based on health behaviour theory [29, 30], had been previously validated, and provided sufficient detail to assess the conceptual basis [50–54].

### Critical appraisal of selected measures

Critical appraisal examined the match between key concepts, selected candidate measures, and SAs because conceptual relevance and socio-cultural alignment were more important than statistical outcomes [55, 56]. Nine items from the Evaluating the Measurement of Patient-Reported Outcomes (EMPRO) [57] were used to assess selected measures for conceptual and measurement model (n = 7), content validity (n = 1), and response burden (n = 1). Two appraisers independently critiqued the first articles, met to discuss results, and reach consensus. Appraisers were selected based on expertise with survey measurement research, and cancer screening research with SAs. The remaining critique of articles was completed by one assessor.

Fifteen potential measures were assessed, and they all met EMPRO criteria for conceptual match (see Table 2). Overall assessment results of “strong recommendation” or “recommendation with potential alterations” were deemed acceptable for inclusion. A final step involved expert consultation of selected measures to retain in the survey.

**Table 2:**
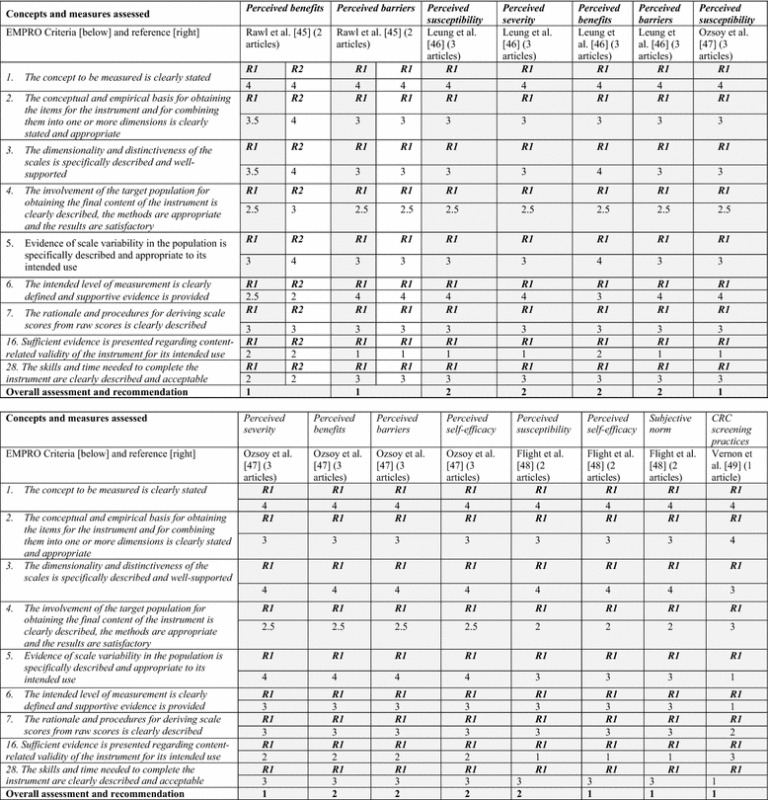
EMPRO tool assessment and scores

(1) Grey highlighted boxes with R1 represent the assessor who critiqued all articles, and R2 is the second assessor who critiqued the first set of articles to ensure consistency of rating; (2) A 4-point Likert scale where 1 is “strongly agree” and 4 is “strongly disagree” is used to determine if instrument developers report required information, suitable methods and findings that reflect good instrument function [57]. The overall assessment and recommendation ranks according to most highly recommended

### Expert consultation

Public health practitioners (*n* = 3) with expertise in cancer screening research and program evaluation were consulted because they worked closely with SAs in Ontario. Ethics approval was obtained from the University of Toronto (#27857) and Brock University (#12-036) Research Ethics Boards. Verbal consent was approved for consultations. Nominal group methods was used [58] to obtain input and endorsement on selected measures to ensure cultural relevance and acceptability for SAs.

The process began with presenting the background and key concepts. Each candidate measure was independently reviewed to ascertain which aligned best to key concepts. Voting cards were used to log selections and provide additional comments. Afterwards, discussion occurred regarding selected measures and potential problems with some items.

Although measures provided good overall matches, selected measures did not fully cover concepts relevant to SAs uncovered in our prior studies [14, 15]. Consequently, three experts in survey measurement and cancer screening were consulted at a separate meeting to provide feedback and ensure complete conceptual coverage in the survey [41]. A total of 17 items [59–65] were added to key concepts for completeness (see Fig. 1). Modifications to items were also required. Informed by our conceptual model, the Colon Cancer Screening Behaviours Survey incorporated 84 items.

### Cross-cultural translation and adaptation and cognitive testing

Cross-cultural translation and adaption into Urdu was conducted [16] following recommended procedures [66, 67]; two individual forward translations; a discussion meeting including a final synthesis report; and, expert committee review. This process resulted in the identification of key issues including missing terms, and difficult or incorrect translation of terms. Thereafter, the survey was cognitively pre-tested with 30 SA immigrants in Canada [16]. General design, culture, and gender related revisions were made, and the survey was further tested with no major problems.

## Conclusions and recommendations

This study adds to prior CRC screening research conducted with SAs in the USA [27, 68] and the UK [25]. Our survey is unique because it was cross-culturally translated and adapted into Urdu, a language chosen because it is widely understood among diverse SAs in the spoken form. In other studies examining CRC screening, surveys targeted English [68] and Hindu and Gujarati speaking SAs [27]. Assessing CRC screening behaviours among SAs requires an adaptation to socio-cultural context. The purpose of our survey is to examine prevalence, beliefs, attitudes, facilitators and barriers to screening among SAs in Canada. Once psychometrically tested, it may be used with English and Urdu speaking SAs in other contexts.

Changes made to published measures were considered necessary to cover key concepts; however, changing survey measures altered measurement properties, which improved measures because of the relevancy to assess CRC screening among SAs; conversely, they could also have been weaken. We believe cognitive testing improved the survey, but it requires further assessment of psychometric properties.

## Limitations

The directed literature review was successful in yielding validated measures; however, because we restricted it to psychometrically tested measures, some untested measures conceptually aligned may have been missed. Nevertheless, modified measures in the survey require psychometric testing. The scoping study findings [14] provided relevant concepts applicable to diverse SAs in the UK, USA and Canada where most studies emerged, while focus group study findings [15] reflected SAs in Canada and thus, may not be representative of those in other contexts. We believe incorporating findings from both studies [14, 15] expanded the breath of understanding CRC screening among SAs in multiple contexts. Consultation capitalized on expertise from individuals working directly with SAs promoting cancer screening and research, and survey measurement; however, only a few experts had international experience.

## Additional file

**Additional file 1.** Literature Search Flow Chart. Presents the process and decisions made at each step of literature search for potential candidate measures for the survey.

## Abbreviations

CRC: colorectal cancer
FOBT: fecal occult blood test
SAs: South Asians
SA: South Asian
UK: United Kingdom
USA: United States of America
HBM: Health Belief Model
TPB: Theory of Planned Behaviour
EMPRO: Evaluating the Measurement of Patient-Reported Outcomes
