# Supplementary material for: Cross-cultural survey development: The Colon Cancer Screening Behaviors Survey for South Asian populations
Source: BMC Res Notes. 2017 Dec 28;10:770. doi: 10.1186/s13104-017-3098-3 (PMC5745603; doi:10.1186/s13104-017-3098-3)
Supplement: Supplementary file 1 — Additional file 1. Literature Search Flow Chart. Presents the process and decisions made at each step of literature search for potential candidate measures for the survey. [file 13104_2017_3098_MOESM1_ESM.docx]

**Comprehensive Literature Search**

Ovid Medline, EMBASE, PsychoINFO, CINHAL, Health and Psychosocial Instruments, Grey literature and review of reference lists using MeSH and free key work search terms*****

**284 citations** excluded

After application of inclusion and exclusion criteria were applied

**426** **citations** returned

**142 potential citations**

- **72** CRC screening
- **50** breast cancer screening
- **20** prostate, cervical, skin cancer or general cancer

**78 citations** excluded

Because they were primarily application studies

**40 citations** excluded

As they did not focus on CRC screening specific measures

**64 potential citations**

Reported on newly developed instrument, previously created measures or modified/adapted measures

**19 citations** excluded

- Lacked alignment with key concept (n=15)
- Limited or no details on conceptual measurement origins or definitions (n=4)

**24 citations reviewed**

Full studies and reports retrieved and reviewed using inclusion and exclusion criteria

**5 citations**

Reporting on measures to be included in the Colon Cancer Screening Behaviours Survey

***Search Terms**: (1) Neoplasms, colorectal neoplasms, breast neoplasms, early detection of cancer, colorectal cancer screening (OR); (2) attitude to health, health attitudes, health behaviour, health beliefs, patient beliefs (OR); (3) reproducibility of results, reliability, test-retest, variance, repeatability, stability, Cronbach’s alpha, Cronbach’s, Congruence (OR). Used Boolean operator AND to combine 1, 2, and 3 search terms.
